# Dysphoria and Irritability—Diagnostic Pitfalls in the Assessment of Interictal Dysphoric Disorder in Epilepsy

**DOI:** 10.3390/jcm10194624

**Published:** 2021-10-08

**Authors:** Agata M. Grzegorzewska, Mariusz S. Wiglusz, Wiesław J. Cubała, Katarzyna Jakuszkowiak-Wojten, Adam Włodarczyk, Joanna Szarmach

**Affiliations:** Department of Psychiatry, Faculty of Medicine, Medical University of Gdansk, 80-952 Gdansk, Poland; mwiglusz@gumed.edu.pl (M.S.W.); cubala@gumed.edu.pl (W.J.C.); k.jakuszkowiak@gumed.edu.pl (K.J.-W.); aswlodarczyk@gumed.edu.pl (A.W.); jszarmach@gumed.edu.pl (J.S.)

**Keywords:** dysphoria, irritability, patients with epilepsy (PWE), interictal dysphoric disorder (IDD), DSM-5 (Diagnostic and Statistical Manual of Mental Disorders)

## Abstract

This article aims to review the concept of epilepsy-specific psychiatric disturbance, Interictal Dysphoric Disorder (IDD), focusing on issues related to its core symptoms and methodological pitfalls. In the psychiatric literature, an epilepsy-specific pleomorphic mood disorder has been long recognized and described as IDD, a condition characterized by eight symptoms, which are grouped into four labile depressive symptoms, two labile affective symptoms, and two specific symptoms. The existence of IDD is still a matter of debate because of several methodological issues. The main features of IDD, such as dysphoria and irritability, lack precise and clear definition. This review article explores the different definitions and approaches towards both terms described in the psychiatric literature and the rationale for modifying the diagnostic process of IDD.

## 1. Introduction

Mood disorders are common in people with epilepsy (PWE) and often go unrecognized and untreated [1,2,3,4]. The symptomatology of mood disorders in epilepsy is often atypical, which makes classification according to standardized psychiatric diagnostic systems, such as the Diagnostic and Statistical Manual of Mental Disorders, Fifth Edition (DSM-5) [5], difficult. In the psychiatric literature, an epilepsy-specific pleomorphic mood disorder has been long recognized and described [6,7]. Blumer et al. [1] coined the term for it, namely Interictal Dysphoric Disorder (IDD). It is a condition characterized by eight symptoms, grouped into four labile depressive symptoms (anergia, depressed mood, insomnia, and pain), two labile affective symptoms (anxiety and fear), and two specific symptoms (euphoric moods and paroxysmal irritability). Those “specific” symptoms refer to a particular group of symptoms of IDD reflected by the term “dysphoria” to highlight the existing outbreaks of irritability, anger, and violent behavior as well as mood changes recurring at specific and regular time intervals [8]. Such episodes of dysphoria occur without outside triggers, consciousness is clear, have a rapid beginning and ending, and recur regularly in an unchanged manner (appear every few days to every few months and last few hours up to two days) [8]. It is suggested that up to 50% of PWE with atypical psychiatric symptomatology corresponding to IDD features are not captured by standardized classificatory systems such as DSM or International Classification of Diseases (ICD) [9].

The concept of IDD first emerged from observations of the German psychiatrists Kraeplin and Bleuler, who noticed a specific pattern of mood and behavioral changes in patients with untreated epilepsy. Symptoms included depressive symptoms mixed up with irritability, fear, anxiety, and euphoric moods, as well as insomnia, anergia, and pain [6,7]. Kraeplin stated that dysphoria is predominant and most common amongst the psychiatric disorders, and dysphoric states present mainly with irritability with or without outbursts of rage. Depressive moods, anxiety, headaches, and insomnia are present, and less often, euphoric moods. Kraeplin noticed that dysphoric episodes appeared both pre-and postictally; however, most commonly were not related to seizures. Similar observations were made by Bleuler [7], who described in the book of psychiatry rich symptomatology of dysphoric states in epileptic patients.

The concept of IDD is still a matter of debate, with some authors suggesting that it is less epilepsy-related than initially presumed [10,11]. Several methodological issues arise when determining the nosological position of IDD as an epilepsy-specific entity. These include differences in study populations, the high ratio of psychiatric comorbidity, and the use of psychometric tools. In many studies, the diagnosis of IDD was established using only self-rating instruments, possibly resulting in biased results [1,2,10,11]. Furthermore, the main features of IDD, such as dysphoria and irritability, lack precise and clear definition. This problem is not only specific to epilepsy but also exists in many psychiatric disorders [12,13,14,15]. Consequently, this review article explores the different definitions and approaches towards both terms described in the psychiatric literature and the rationale for modifying the diagnostic process of IDD, thus being able to address these methodological issues. It is important to emphasize that much of the data discussed here are preliminary and therefore need to be validated in future studies. Nevertheless, in order to disregard or acknowledge the nosological identity of IDD, future studies are necessary, and a comprehensive diagnostic model for IDD needs to be designed and validated. 

The authors searched Google Scholar/Pubmed using the keywords: dysphoria, irritability, patients with epilepsy (PWE), interictal dysphoric disorder (IDD), DSM-5 (Diagnostic and Statistical Manual of Mental Disorders). Only papers in English have been considered.

## 2. Impact of IDD and Psychiatric Comorbidity on Quality of Life and Risk of Suicide

Recognition of IDD and other comorbid psychiatric disorders in PWE is of vital importance in clinical practice. If it remains unrecognized, it may negatively affect the course and treatment of epilepsy and contribute to a significant decrease in quality of life. A study on Danish epilepsy outpatients revealed the prevalence of IDD for 19%. Moreover, patients with IDD and additional psychiatric comorbidity had significantly higher seizure frequency, higher level of side effects to the antiepileptic treatment, and lower quality of life, both when compared to patients with normal screening and patients with IDD as the only comorbidity [10]. The authors suggest that the health status of epilepsy patients with IDD seems to depend on other psychiatric comorbidities more than on IDD [10]. Results of another study point out depression as a powerful predictor of Quality of Life (QoL) that may be inadequately prioritized in the management of epilepsy [16]. Remarkably, 37% of epilepsy patients with IDD revealed suicidality as assessed by the Mini-International Neuropsychiatric Interview (MINI). However, the authors suggest that the severity of suicidality is more dependent on the presence of depression or other psychiatric comorbidities than IDD itself [10]. Epilepsy patients have an increased risk of committing suicide even in the absence of psychiatric comorbidity, but the highest risk has been found in patients with epilepsy and comorbid psychiatric disease [17]. Recently, Hesdorffer et al. [18] found that epilepsy is associated with an increased risk of psychiatric disorders and suicide even before the epilepsy diagnosis. Suda et al. [19] raise the issue of suicidality in patients with Localization Related Epilepsy (LRE), IDD, and concomitant psychiatric disorders. Compared to patients without IDD, patients with IDD had extremely high comorbidity rates for mood disorders (68.0% vs. 20.4%), anxiety disorders (52.0% vs. 12.6%), and psychotic disorders (48.0% vs. 10.7%). The authors concluded that IDD was significantly associated with comorbid psychiatric disorders (PDs), earlier-onset epilepsy, refractory Complex Partial Seizures (CPS), psychotropic drug use, subjective adverse effects of anti-epileptic drugs (AEDs), and subjective depression. Moreover, IDD added an extreme psychosocial burden. Alternately, patients with IDD may be susceptible to various PDs, or IDD may be specific to patients with epilepsy who have comorbid multiple PDs.

## 3. Assessment of IDD

**Figure 1:**
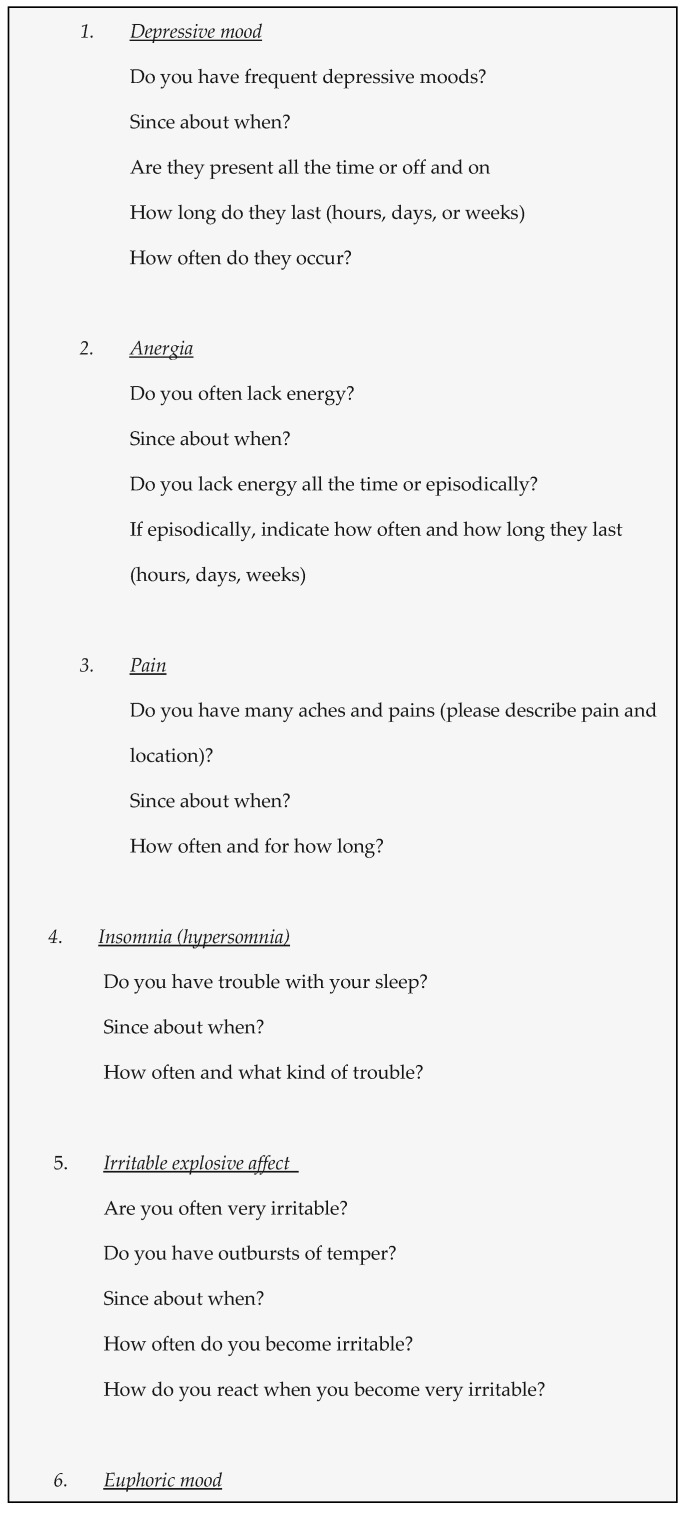

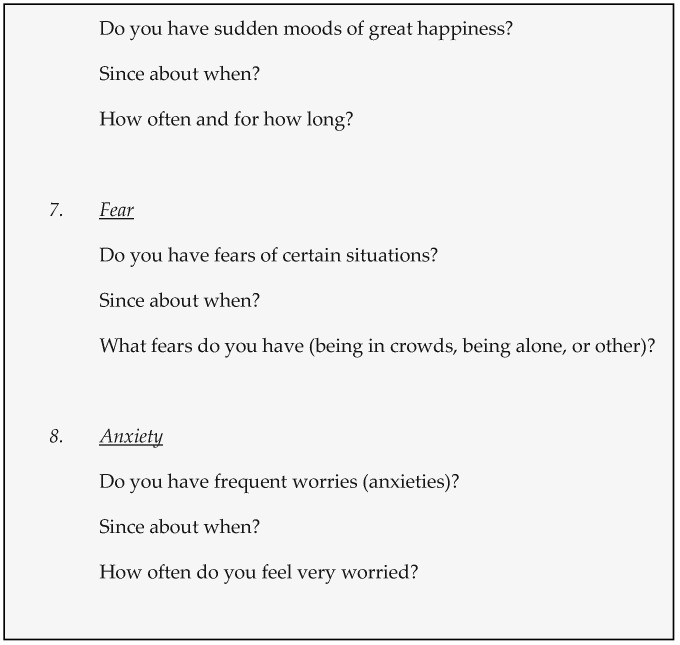
Set of questions from the Seizure Questionnaire (modified from: Blumer et al. [1]).

Blumer et al. [1,8,20,21] developed a set of questions incorporated into the Seizure Questionnaire (SQ), which aims to assess 8 key symptoms of IDD during the last 12 months (Figure 1).

IDD is diagnosed when at least three of the eight core symptoms are confirmed to be clinically significant. In order to improve recognition of IDD in epilepsy, Mula et al. [2,8,9] developed Interictal Dysphoric Disorder Inventory (IDDI), a 38-item self-questionnaire specifically designed to investigate the core symptoms of IDD (anergia, pain, insomnia, fear/panic, anxiety, depression, euphoria, irritability). The IDDI comprises eight main inquiries with further auxiliary questions and six additional questions that investigate the time course of the disorder, duration of dysphoric symptoms, and their associations with seizures or antiepileptic drug therapy. The symptoms are evaluated in terms of presence, frequency, severity, and global impairment. To diagnose IDD, at least three out of eight key symptoms must be present and of at least “moderate” or “severe” severity and causing “moderate” or “severe” distress. The IDDI is specific for symptoms of IDD in patients with epilepsy and cannot be used to discriminate between unipolar depression and IDD. The instrument showed good internal consistency, an acceptable sensitivity, and excellent specificity [2,9]. The main difference from SQ is that originally, after completing the self-rated Seizure Questionnaire, an examiner reviewed all the data for completeness, accuracy, and recognition of the clinical significance of each key symptom, whereas IDDI relies solemnly on self-evaluation.

Assessing whether any clinical manifestation of a new nosological entity is specific to the point that it differs from other disorders is a complex process, and IDD with its symptoms conglomerate is not an exception. Feighner et al. [22] stated, “In general, the first step is to describe the clinical picture of the disorder”. This may be a single striking clinical feature or a combination of clinical features thought to be associated with one another. In epilepsy, irritability, together with other labile affective symptoms, identifies a clinical symptom cluster of IDD that is reflected by the term “dysphoria” in order to stress the periodicity of mood changes and the presence of outbursts of irritability and aggressive behavior [1,2,8,9,20,21]. However, the terms dysphoria and irritability that best describe IDD are generally poorly defined and, therefore, measured inconsistently. For example, irritability in IDDI is defined as feeling “irritable, experience bad temper, or fly off the handle easily over little things from time to time”, which may be interpreted not only as irritable mood but also as anger or even aggression.

## 4. Mood Dimensions

Mental disorders are classified in the diagnostic classification systems (DSM/ICD) as categorical criteria (i.e., psychiatric disorder entities are either present or absent), so it is difficult to assign a particular psychopathology adequately to a specified nosological entity. Research data indicates that many psychopathological symptoms that belong to mental disorders occur on a spectrum and may be present to some degree even in the “normal” population [23,24]. Moreover, it is not possible to clearly and reliably set the boundaries between given disorders, or a disorder and normality. Considering a variety of psychiatric symptoms, atypical manifestation or subthreshold forms of a particular disorder may not be precisely identified with diagnostic classification criteria or overlap with another disorder’s criteria [23,24]. All of this together should be considered when, for instance, affective symptoms such as dysphoria or irritability are assessed in IDD.

### 4.1. Dysphoria

In psychiatry, dysphoria is a psychopathological concept that has been used to characterize a wide range of psychopathology. Dysphoric mood is present in Major Depressive Disorder (MDD), Mixed States of Bipolar Disorder (DSM-5 abandoned the criteria “mixed episode” and created the new specifier “with mixed features” instead), also the newest classification of psychiatric disorders DSM-5 [5] provides diagnostic criteria for a premenstrual dysphoric disorder and gender dysphoria. Moreover, dysphoria represents a feature of personality disorder (‘hysteroid dysphoria’), is used in the context of side effects of antipsychotic medications (‘neuroleptic dysphoria’), or after long-term use of antidepressants (Antidepressant-associated chronic irritable dysphoria—ACID and ‘tardive dysphoria’), is an important dimension of Post-Traumatic Stress Disorder (PTSD) [12,13,25,26,27] and borderline personality disorder. Of note, the clinical manifestation of IDD is very similar to that of Premenstrual Dysphoric Disorder (PDD), which is characterized by a variety of emotional states (irritability, anger, depressed mood, anxiety, and ‘marked affective lability), with only one of them having to be present to make the diagnosis. The characteristics of both the interictal and the premenstrual dysphoric disorders include symptoms of mood and anxiety presenting in an intermittent pattern, together with heightened irritability that ranges from increased temper to explosive rage [28]. Some authors also have conceptualized dysphoria as a “third dimension” in addition to mania and depression and defined it as “a morose, tense, and irritated mood”. Bertschy et al. [29] defined dysphoria as three of the following four symptoms: inner tension, irritability, aggressive behavior, and hostility.

Dysphoria appears to be an unstable and unpredictable “entity” and is a broader concept in which irritability is only one aspect. However, the current confusion regarding the concept of dysphoria gets even more complicated when we also look at the inconsistency in definitions of irritability across psychiatric diagnoses.

### 4.2. Irritability

Irritability is one of the prominent transdiagnostic constructs in the DSM-5, ranging across 15 disorders, including Mood Disorders, Trauma- and Stressor-Related Disorders, Disruptive, Impulse-Control and Conduct Disorders, Substance-Related and Addictive Disorders, and Personality Disorders [5]. Although it is occasionally wrongly equated with dysphoria, major confusion has existed in discriminating irritable mood from anger or irritable behavior from aggression or hostile behavior [13,14]. This overlap in the conceptualizations of irritability, anger, and aggression is also apparent in IDDI.

Toohey et al. [14], based on an excellent review of the data on irritability, discussed a more precise definition that differentiates it from related constructs. According to this new definition, irritability is: (a) a mood, (b) associated with partial agitation, (c) associated with increased sensitivity to sensory stimuli, (d) non-cognitively mediated, (e) a lowered threshold for provoking stimuli, (f) followed by anger and aggression, and (g) directly linked to physiological/biological changes. The results of two recent studies of IDD in epilepsy provide some information about symptom frequency as measured with IDDI [10,11]. Irritability was found in a higher frequency in patients with IDD and additional psychiatric disorders compared to PWE without IDD or any other comorbid psychiatric disorder. The authors concluded that these symptoms are not specifically prominent among PWE (with and without IDD) [11]. Apart from these studies, irritability has been studied in epilepsy mostly as a symptom of its own. In order to measure irritability and related constructs, scales such as Irritability Questionnaire, Penn State Worry Questionnaire, Intolerance of Uncertainty Scale, Aggression Questionnaire (AQ) were used [30,31]. Another study aimed to analyze the psychometric properties of a new Italian instrument of irritability in PWE (I-Epi) [31]. Moreover, the findings of one study using I-Epi suggest that almost 60% of epilepsy patients exhibited at least a moderate level of irritability [32]. Besides, emerging evidence suggests that symptoms of bipolar disorder (BD) such as mood instability, mixed irritability, and manic episodes are not uncommon in patients with epilepsy [33]. Theoretically, epilepsy and BD share some clinical and neurobiological features (episodic course, often becomes chronic, ‘kindling’ phenomenon, AEDs with mood-stabilizing properties). Recent data suggest that irritability and other symptoms of BD could be manifestations of epilepsy-specific psychiatric disturbances such as preictal dysphoria, postictal mania, interictal dysphoric disorder, and treatment-emergent adverse effects of medications [33]. According to Mula et al. [2], IDD belongs more to the bipolar than the unipolar spectrum of affective disorders. It is possible that BD is underdiagnosed in PWE because it has been much less investigated than unipolar depression in this population [34,35].

### 4.3. Periictal Mood Changes

It is worth noting that IDD symptoms may, in fact, be related to peri-ictal manifestation. In their study, Mula et al. [36] aimed to investigate clinical correlates of IDD and seizures in PWE and make a distinction between IDD and Peri-ictal Dysphoric Disorder (PDD). About 50% of patients primarily diagnosed with IDD presented symptoms with a clear-cut relationship with epileptic seizures that occurred either preictally or postictally or reported symptoms that were habitually related only to seizures [36]. A study by Gaitatzis et al. [37] revealed that one-third of patients with temporal lobe epilepsy (TLE) report prodromal symptoms preceding secondarily generalized tonic–clonic seizures [37]. Also, prodromal mood changes may occur hours to days before a seizure and are often relieved by the convulsion [38]. A cross-sectional study in tertiary referral centers in Europe pointed out that around 13% of patients may experience irritability, dysphoria, or depressed mood preceding seizures [36]. It is estimated that around 12% of patients with TLE may present isolated peri-ictal dysphoric symptoms without interictal chronic symptoms [36]. In contrast to preictal psychiatric manifestations, postictal mood changes are rarely recognized by clinicians. In a case series of presurgical patients, up to 18% reported postictally at least five symptoms of depression lasting more than 24 h, up to 22% presented manic symptoms often with associated hallucinations or delusions [39], and 45% reported postictal anxiety [39]. Considering the complexity of symptoms of IDD or PDD, it is possible to over-diagnose psychiatric disorders or misdiagnose behavioral problems. A cross-sectional study of patients recruited in two epilepsy centers in Europe revealed that a diagnosis of bipolar disorder may be overestimated in epilepsy if peri-ictal mood changes are not correctly identified [40]. In fact, out of the 11.8% of DSM-based diagnoses of bipolar disorder, only 1.4% could be considered as a “pure” psychiatric diagnosis because, in all other cases, manic/hypomanic symptoms were temporally related to seizures occurring either postictally or preictally [40]. The peri-ictal dysphoric disorder represents a number of behavioral symptoms that may occur before or after the seizure and is likely to give atypical symptomatology of mood disorders in epilepsy. Of note, such symptoms may be subtle, often fail to meet DSM criteria for affective symptoms/disorders, and are difficult to identify by clinicians [9]. It is important to distinguish IDD from PDD as both entities need different therapeutic approaches. In IDD, specific psychiatric treatment should be applied, such as pharmacological or psychotherapeutic interventions because symptoms may be chronic and unremitting, while in the latter, controlling seizure is predominant for the management of seizure-related affective manifestations [1,8,36]. This implicates a necessity of a careful assessment of seizure-based behavioral symptoms and alertness to atypical manifestations of psychiatric disorders as part of routine neurological examination.

### 4.4. Emotional Dysregulation

Increasing evidence of a strong relationship between emotional dysregulation and many neuropsychiatric conditions has been observed. There is also a question of the direction of causality, i.e., whether some forms of emotional dysregulation play a vital role in the development of certain mental disorders or represent their consequence [41]. In epilepsy, such emotional instability could lead to autonomous fluctuations of mood that develop later into pleomorphic, intermittent symptomatology often seen in PWE. Consequently, we should reconsider our understanding of psychopathology in PWE and not only as a part of DSM-defined mood and anxiety disorders or epilepsy-specific phenomena. Of note, some studies suggested it could be possible that an underlying dysphoric/irritable temperament somehow shapes interictal symptomatology in epilepsy [9,42]. Future research should examine the role of emotional regulation in the development of psychiatric disorders in PWE.

## 5. Diagnostic Difficulties of IDD in Clinical Practice and Therapeutic Approach

The lack of a precise definition of irritability and constructs related to dysphoria may undermine the diagnostic ability of IDDI, which is so far the only tested and validated tool for recognition of IDD. The clinical presentation of Interictal Dysphoric Disorder, with its intermittent and pleomorphic symptomatology, is well described in the literature; however, due to lack of precise definitions and tools, it is difficult to establish its nosological position. In this questionnaire, irritability is defined in a way that it comprises multiple types of symptoms, making it hard to score consistently. For example, if someone has an irritable mood, but all other constructs related to anger or impulsive behavior are absent, it is not clear whether the rating should be weighted towards the presence of irritability. Constructs such as irritability, anger, aggression, or other impulsive behavior should be measured separately. There have been concerns and doubts raised regarding IDD as a separate nosological entity. Zinchuk et al. [43] in their study did not confirm the specificity of IDD for epilepsy. They suggest that the presence of IDD symptoms may be associated with a more severe course of MDD and significant anxiety distress. There were no differences found in the prevalence of IDD between PWE with MDD and people with MDD alone. Based on the results of their study, Amiri et al. [10] cast in doubt the existence of IDD as a nosological entity and emphasize that reliable tools for its identification are lacking. Other findings that challenge the evidence for IDD as epilepsy-specific are those by Labudda et al. [11]. The authors indicate that IDD occurs with the same frequency and the same symptom cluster in a purely psychiatric sample. The study mentioned the majority of PWE diagnosed with IDD had or have had a psychiatric diagnosis, mainly depression and anxiety disorders, suggesting a strong overlap of IDD and DSM Axis I diagnoses. The authors point out the possibility of the redefinition of IDD in the future by conceptualizing it as a disorder that should only be diagnosed in patients with no psychiatric comorbidities.

However, given the fact that IDD may be significantly associated with comorbid PDs in epilepsy, such as mood or anxiety disorders [2,19], it is worth reflecting on whether IDD can be considered as a separate nosological entity that can be diagnosed only in PWE with no concurrent psychiatric disorders. Moreover, it is necessary to bear in mind that the accuracy of psychiatric diagnosis in epilepsy may be affected by side effects of antiepileptic drugs (AED) as well as peri-ictal symptomatology [44]. In order to determine whether IDD is a nosologically independent disorder, future studies should focus on the development and evaluation of updated diagnostic tools for recognition of IDD.

### 5.1. Screening for Mood Disorders in Epilepsy

It is important to screen for mood disorders as psychiatric comorbidity in epilepsy is common. The Neurological Disorder Depression Inventory for Epilepsy (NDDI-E) is a screening tool for depression that was developed for use specifically in patients with epilepsy. It comprises 6 statements about the past 2 weeks that are scored between 1 (never) and 4 (always/often), with a minimum score of 6 and a maximum of 24. In the original version, a score above 15 has been shown to have a high predictive value for major depression [45]. However, the utility of screening instruments in detecting mood disorders cannot be overestimated as the only reliable tools. They help identify patients who need further attention and inquiry, and this must be accompanied by an assessment of seizure-based behavioral manifestations [8]. In-depth inquiry towards psychiatric symptoms can be performed with SQ or IDDI to detect clinical features of IDD or PDD; The Seizure Severity Questionnaire (SSQ) can also be used to assess the severity and bothersomeness of seizure components and thereby evaluate the existence of potential depressive symptoms. SSQ investigates recovery from physical, emotional, and cognitive effects of seizures and allows defining which component was most affected by comorbid depression. Compared to non-depressed people experiencing similar types of seizures, people with clinical symptoms of depression report higher levels of subjective severity and bother from seizures, and also more difficulties in overall seizure recovery [46].

### 5.2. Therapeutic Approach for IDD

Considering the rich and atypical symptomatology of mood disorders in epilepsy, it is necessary to dissect out peri-ictal manifestations from interictal ones as both of them have relevant but different implications in terms of the course of the disorder, course of treatment, and prognosis [8,9]. In IDD, pharmacological interventions are usually indicated when at least three of the eight key symptoms of dysphoric disorder are present [1]. Selective serotonin reuptake inhibitors (SSRIs), tricyclic antidepressants (TCAs), and neuroleptics are favored, and benzodiazepines (BZD) or psychostimulants are also used [1]. The medication chosen depends on the clinical features of IDD. If anergia and hypersomnia predominate, activating antidepressants, such as fluoxetine or imipramine, are the first choice. On the other hand, if depressive moods are combined with insomnia and anxiety, SSRIs and tricyclics with sedative properties bring good effects. Amongst SSRIs, paroxetine, sertraline, citalopram, and fluoxetine may be chosen, augmented with TCA. If the patient does not respond properly to the treatment, the action of antidepressants may be enhanced by small doses of risperidone (1–2 mg) or olanzapine (5 mg), as well as methylphenidate or benzodiazepines (alprazolam) [1]. At each therapeutic intervention (ex. First visit, after 1 month, after 2 months), the status of each of the eight symptoms of the dysphoric disorder is assessed. Usually, response to treatment is achieved for all the symptoms of IDD and can be expected when the therapeutic dose is reached. The mechanism of action of the antidepressant drugs is different in IDD and in DSM classified depressive disorders—the drugs are effective at smaller doses, act rapidly and on a broad spectrum of symptoms, not only those from the labile and affective group but also for interictal psychotic symptoms (such as hallucinations, delusions, and paranoid ideas). In these patients, double antidepressant treatment is augmented with antipsychotic medication [1].

## 6. Conclusions

The following limitations of our review should be considered. This is a non-systematic review; therefore, the conclusions are not definite. Much of the data discussed here are preliminary and need to be validated in future studies.

In clinical practice, the recognition of IDD is of vital importance. As psychiatric manifestations of IDD are often atypical and chronic, it is important to perform a careful assessment of IDD symptoms as part of a routine neurological examination and implement psychiatric treatment if needed. Chronicity of affective symptoms, including mood changes, irritability, intermixed with insomnia, fear, and anxiety, may result in poor compliance in epileptic treatment (e.x. missing doses of recommended medications), bad seizure control, and further decompensation of the mental state with high suicide risk. It is worth noting that IDD may have high comorbidity with other psychiatric disorders. Recognition and treatment of psychiatric disorders in epilepsy may have implications for the course of both medical conditions, responses to treatment and health outcomes, and hence, the quality of patient’s life.
